# Impact of open-irrigated radiofrequency catheter with contact force measurement on the efficacy and safety of atrial fibrillation ablation: a single-center direct comparison

**DOI:** 10.1007/s10840-022-01316-8

**Published:** 2022-07-30

**Authors:** Simon Schlögl, Klaudia Stella Schlögl, Philipp Bengel, Leonard Bergau, Helge Haarmann, Eva Rasenack, Gerd Hasenfuss, Markus Zabel

**Affiliations:** 1 Department of Cardiology and Pneumology, Heart Center, University Medical Center, Robert-Koch-Str. 40, 37075 Göttingen, Germany; 2 DZHK (German Center for Cardiovascular Research), Partner Site Göttingen, Göttingen, Germany

**Keywords:** Atrial fibrillation, Pulmonary vein ablation, Contact force sensing, Catheter ablation

## Abstract

**Background:**

In atrial fibrillation (AF) patients, catheter ablation of pulmonary veins (PVI) is the most effective therapeutic option to maintain sinus rhythm. To improve successful PVI, contact force–sensing (CF) catheters became routinely available. Previous studies did not clearly show superior clinical efficacy in comparison with non-CF catheters.

**Methods:**

We investigated consecutive patients, who underwent index PVI for AF at our hospital between 2012 and 2018. Three hundred and fifty-four patients were ablated without CF. After availability of CF catheters in 2016, 317 patients were ablated using CF. In case of crossover between the groups, follow-up was censored. The primary endpoint was any documented atrial tachycardia (AT) or atrial fibrillation > 30 s after a 3-month blanking period. Secondary endpoints were procedural characteristics and periprocedural complications.

**Results:**

There was no significant difference between the groups at baseline except hyperlipidemia. After 365 days of follow-up, 67% of patients in the CF group remained free from AF/AT recurrence compared to 59% in non-CF group (*P* = 0.038). In multivariable Cox regression analysis, non-CF ablation was an independent risk factor for AF recurrence besides age and persistent AF. Total fluoroscopy time (15 ± 7.6 vs. 28 ± 15.9 min) and total procedure time (114 ± 29.6 vs. 136 ± 38.5 min) were significantly lower for CF-guided PVI (*P* < 0.001). Complication rates did not differ between groups (*P* = 0.661).

**Conclusions:**

In our study, the AT/AF recurrence rate and pulmonary vein reconnection rate is lower after CF PVI with a similar complication rate but lower total procedure time and total fluoroscopy time compared to non-CF PVI.

## Introduction

Radiofrequency catheter ablation of the pulmonary veins (PVI) has emerged as the primary standard of care in patients with drug-refractory atrial fibrillation (AF) and has been proven more effective than antiarrhythmic agents in maintaining sinus rhythm on a mid- to long-term basis [1, 2]. The population of patients eligible for PVI is steadily expanding, PVI is applicable in patients with paroxysmal, persistent, and long-persistent AF and its indication is guided primarily by the severity of patient’s symptoms [1]. In our center, PVI is a routine procedure for drug-refractory symptomatic AF since 2006. Later on, PVI has become the first choice of AF treatment for patients with symptomatic AF in our center [1]. The efficacy of PVI is mainly dependent on the formation of durable transmural atrial lesions that maintain conduction block between ablated sites and surrounding left atrial tissue [3]. The contact force (CF) exerted by the catheter tip on atrial tissue is a crucial determinant of effective lesion creation [4]. Low CF is associated with early electrical reconnection while excessive CF increases the risk of steam pops and cardiac perforation [4, 5]. Traditionally, catheter tip-tissue contact was indirectly assessed by using indirect markers such as electrogram amplitude, impedance change, and electrode temperature, which do not consistently correlate with CF [6–8]. To address this issue, CF-sensing catheters were developed that directly measure tissue contact and provide real-time data to guide operators performing PVI [9, 10]. While most of the previous observational studies (OS) (and subsequent meta-analyses of such studies) [11–13] have demonstrated a varied benefit from CF-guided AF ablation, recent randomized trials (RCT) failed to show a benefit [9, 10, 14–20]. Hence, despite enthusiastic uptake and widespread adoption, the very impact of CF-sensing technology on clinical outcomes still remains unclear [13]. Therefore, we conducted the present single center observational study to assess the safety and efficacy of CF-guided vs. non-CF-guided open-irrigated PVI for AF with a follow-up of 1 year.

## Methods

### Patient’s characteristics

A total of 671 consecutive patients with AF were included between January of 2012 and December of 2018. Thereof, 317 consecutive patients underwent open-irrigated CF-sensing catheter ablation for AF (Thermocool® SmartTouch ™ Surround Flow ™, Biosense-Webster, Diamond Bar, USA) between 2016 and 2018. We compared these patients with 354 patients who underwent ablation with an open-irrigated catheter without CF-sensing (Thermocool® Surround Flow ™, Biosense-Webster, Diamond Bar, USA) between 2012 and 2016. Baseline characteristics of the two groups are shown in Table 1. In total, 63% of the patients presented with persistent AF at baseline.

**Table 1:** Baseline

	Non-CF (354)	CF (317)	*P* value
Gender (male)	223 (63.0%)	191 (60.3%)	0.633
Age (years)	62.8 ± 10.0	64.4 ± 10.7	0.052
BMI (kg/m^2^)	29.0 ± 5.2	29.2 ± 5.3	0.605
Arterial hypertension	270 (76.3%)	252 (79.5%)	0.224
Coronary artery disease	75 (21.2%)	74 (23.3%)	0.515
COPD	25 (7.1%)	24 (7.5%)	0.882
DCM	13 (3.7%)	12 (3.8%)	1.000
Sleep apnea	24 (6.8%)	23 (7.3%)	0.880
Hyperlipidemia	146 (41.2%)	165 (52.1%)	**0.004**
Smoking	67 (18.9%)	65 (20.5%)	0.627
Diabetes	47 (13.3%)	45 (14.2%)	0.736
Left atrial diameter (mm)	43.3 ± 7.0	42.5 ± 7.9	0.211
LVEF (%)	53.1 ± 5.2	52.6 ± 6.5	0.250
Paroxysmal AF	139 (39.3%)	112 (35.4%)	0.230
Persistent AF	215 (60.7%)	205 (64.6%)	0.230
CAAP-AF score[38]	6.0 ± 2.1	5.8 ± 2.3	0.492

*BMI*, body mass index; *COPD*, chronic obstructive pulmonary disease; *DCM*, dilatative cardiomyopathy; *LVEF,* left ventricular ejection fraction; *AF,* atrial fibrillation

### Ablation procedure

All patients gave informed consent prior to the ablation procedure. In all subjects, left atrial (LA) thrombi were excluded by transesophageal echocardiography, and LA anatomy was acquired by contrast-enhanced high-resolution thoracic computer tomography prior to the procedure. All ablation procedures were performed with conscious sedation using intravenous sufentanil, midazolam, and/or propofol under continuous monitoring of blood pressure and oxygen saturation. For the electrophysiological procedure, all catheters were advanced via the femoral veins. A 6F steerable decapolar catheter (Bard Dynamic Tip, Bard Inc., Lowell, MA, USA) was positioned in the coronary sinus. After fluoroscopically guided double transseptal puncture (TSP), an SL1 sheath (St. Jude Medical, St. Paul, MN, USA) and an Agilis® deflectable sheath (St Jude Medical, St. Paul, MN, USA) were advanced into the LA. In the CF group an open-irrigated, mapping, and contact force–sensing ablation catheter (Thermocool® SmartTouch ™ Surround Flow ™, Biosense-Webster, Diamond Bar, USA) was advanced through the sheath into the LA, whereas in the non-CF group an open-irrigated, mapping, and ablation catheter (Thermocool® Surround Flow™, Biosense-Webster, Diamond Bar, USA) was used. A circular mapping catheter (Lasso®, Biosense Webster, Diamond Bar, CA, USA) was positioned within the pulmonary vein (PV) ostium to monitor electrical activity during ablation and to verify electrical PV isolation. Intravenous heparin was administered immediately after the TSP to maintain an activated clotting time (ACT) of 300–350 s throughout the procedure. Patients presenting with persistent atrial fibrillation underwent electrical cardioversion prior to mapping and ablation. Left atrial angiography was performed prior to generation of a 3D electroanatomic model of the left atrium and the PV ostia using a three-dimensional mapping system (Carto® Biosense Webster). To assure an accurate 3D model acquisition, respiratory gating was performed. The resolution level of the 3D system during anatomy acquisition in the “fast anatomical mapping (FAM)” mode was set to a minimum of 15. RF current was applied with 40 W on the anterior left atrium (LA) and 30 W at the posterior LA wall with a generator (Stockert, Biosense Webster) in a power-controlled mode with an upper temperature limit of 45 °C and a standardized, power-dependent irrigation rate. In the non-CF group, RF current was applied for 30–60 s until local electrogram amplitude was reduced by 80%. An interlesion distance of ≤ 6 mm was aimed for. In the CF group, contact force was continuously monitored. According to manufacturer’s protocol a contact force of 5–20 g was targeted during ablation. A force time integral (FTI) with an aim of 330 g s was used to determine acceptable lesions. Excessive tissue contact force (> 50 g) was visually indicated for safety considerations. Endpoint of the ablation procedure was the electrical isolation of all PVs defined as bidirectional conduction block. This was verified by the lasso catheter and a careful and repeated mapping for residual potentials around the entire circumference of the PV ostia, and pacing from multiple sites within the circumferential line. All pulmonary veins were examined at the end of the procedure resulting in waiting periods of longer than 20 min for the LSPV and LIPV and approximately 5 min for RSPV and RIPV. In the case of persistent AF, additional ablation lines were considered during the repeat ablation at the discretion of the operator. All procedures were performed by the same experienced operators.

### Follow-up

After hospital discharge, patients were followed in our outpatient clinic and a 96 h Holter-ECG was performed after 3, 6, and 12 months after the index procedure. At each visit, subjects were asked for symptoms, documented arrhythmia recurrences, and current medication was assessed; 139 (21%) of the patients had an implanted cardiac device, which was interrogated in every visit. Furthermore, all patients were advised to present themselves immediately in case of symptoms suggestive for arrhythmia recurrence and obtain ECG documentation. An electrical cardioversion was performed prior to discharge in case of detected AF/atrial tachycardia (AT) recurrence post-interventional for AF episodes lasting longer than 6 h. Furthermore, in some cases, antiarrhythmic drugs (AADs) (flecainide, propafenone, dronedarone, amiodarone) were continued for the next 3 months (blanking period) with termination of the antiarrhythmic medication after the blanking period. A documented AF/AT episode lasting longer than 30 s after the blanking period was considered a recurrence. Additional diagnostic information (e.g., echocardiogram, chest X-ray/computer tomography) was acquired if symptoms were suggestive of procedure-related complications (e.g., pericardial effusion, pulmonary vein stenosis). In the case of crossover between groups, follow-up was censored.

### Statistical analysis

Variables are expressed as mean ± standard deviation (SD) if normally distributed, or as percentage or median value with 25^th^ and 75^th^ percentiles interquartile range (IQR). Differences in the frequency of characteristics were assessed by independent samples Student’s *t*-test for continuous variables. Chi-square statistic (or Fisher’s exact test if applicable) was used for discrete/categorical variables. Probability of AF recurrence was based on the time to first AF recurrence after the index procedure determined by Kaplan–Meier analysis with Mantel-Cox (log-rank) test. Time to first AF recurrence was plotted as a Kaplan–Meier curve. If a crossover between CF and non-CF groups occurred, follow-up was censored. A Cox proportional hazards model with multiple variables was performed to identify predictors of AF recurrence in a multivariable analysis at follow-up. All tests were performed with a two-tailed significance level of 0.05. We used SPSS 23.0 (SPSS, Inc.) for data storage and analysis.

## Results

### Baseline, procedure

At baseline, there were no significant differences between the groups except the number of patients with hyperlipidemia (Table 1). Three hundred and fifty-four patients in the CF group underwent a mean of 1.2 ± 0.5 procedures, in the non-CF group 317 patients had 1.2 ± 0.5 procedures (*P* = 0.325) resulting in a total of 828 ablation procedures. A complete electrical isolation of all PVs was achieved in 100% of the CF-guided cases (386 out of 386 ablations) and in 99% of the non-CF cases (438 out of 442 ablations) (*P* = 0.128). The ablation characteristics and follow-up data of both groups are summarized in Table 2. In the CF group, total procedure time and total fluoroscopy time were significantly lower compared to the non-CF group. There was no difference in the radiofrequency application time between the groups.

**Table 2:** Ablation characteristics and follow-up

	Non-CF (354)	CF (317)	*P* value
Procedure time (min)	136 ± 38.5	114 ± 29.6	** < 0.001**
Fluoroscopy time (min)	28 ± 15.9	15 ± 7.6	** < 0.001**
Radiofrequency time (sec)	1228 ± 689.7	1219 ± 550.8	0.853
Major peri- and post-procedural complications (%)	10 (2.3%)	11 (2.9%)	0.661
Total number of reablations	1.2 ± 0.5	1.2 ± 0.5	0.325
Lost to follow-up (%)	22 (5%)	23 (6%)	0.542

### Safety

Out of 828 procedures, 10 out of 442 ablations (2.3%) in the non-CF group and 11 out of 386 ablations (2.9%) in the CF group, experienced major peri-or post-procedural complications (*P* = 0.661). In total, 5 patients (0.6%) had an accidental aortic puncture when the transseptal puncture was performed. All of the group-specific complications are listed in Table 3.

**Table 3:** Safety endpoints

Safety endpoint	Non-CF (442)	CF (386)	*P* value
Pericardial effusion	5 (1.1%)	3 (0.7%)	n.s
Post-procedural stroke	0 (0%)	1 (0.2%)	n.s
Phrenic nerve palsy	0 (0%)	2 (0.5%)	n.s
Femoral arteriovenous fistula requiring surgical intervention	3 (0.6%)	1 (0.2%)	n.s
Post-procedural groin bleeding requiring surgical intervention	1 (0.2%)	1 (0.2%)	n.s
Atrio-esophageal fistula	0 (0%)	0 (0%)	n.s

*n.s.*, non-significant

### Efficacy

**Fig. 1:**
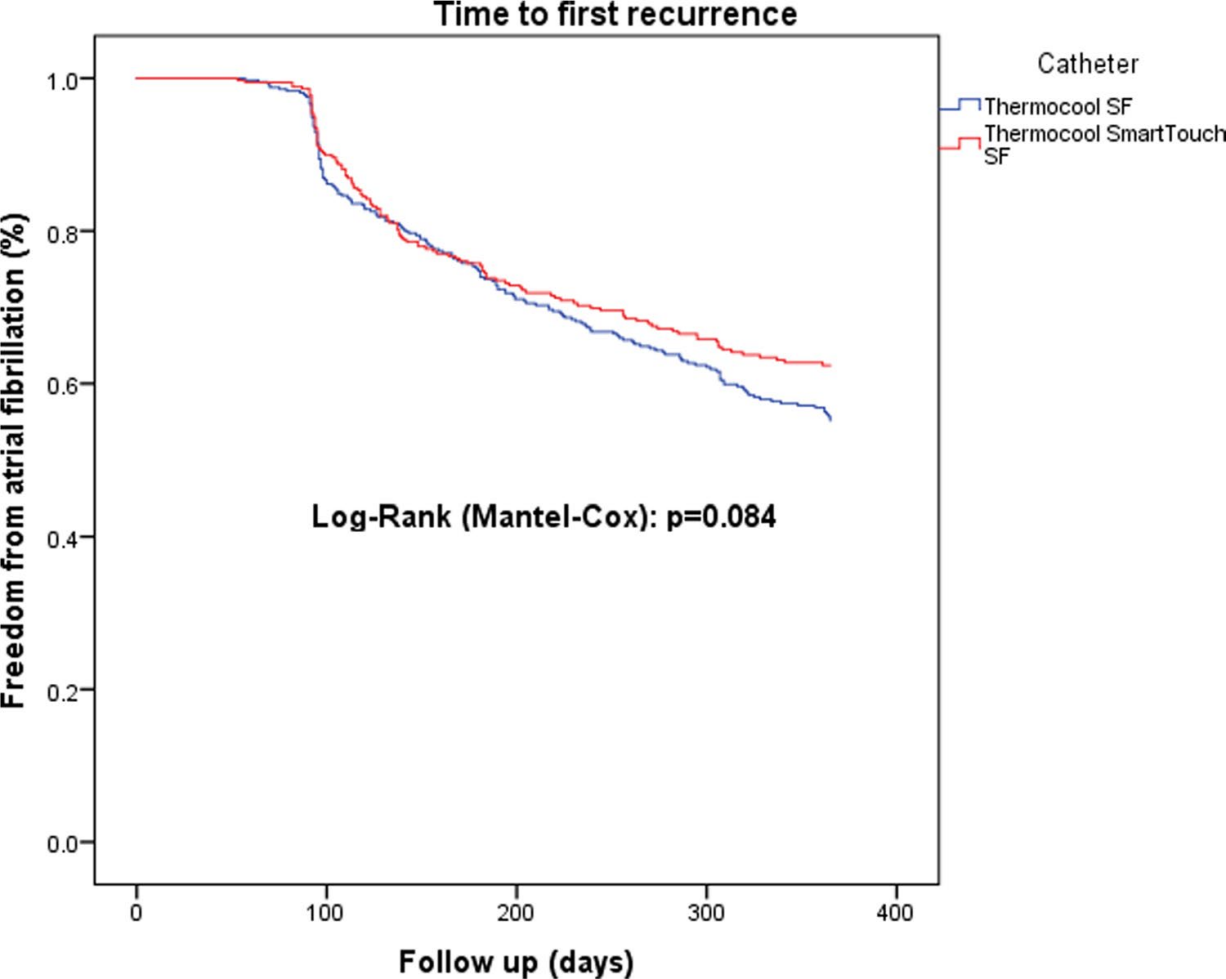
Time to first recurrence after index ablation

**Fig. 2:**
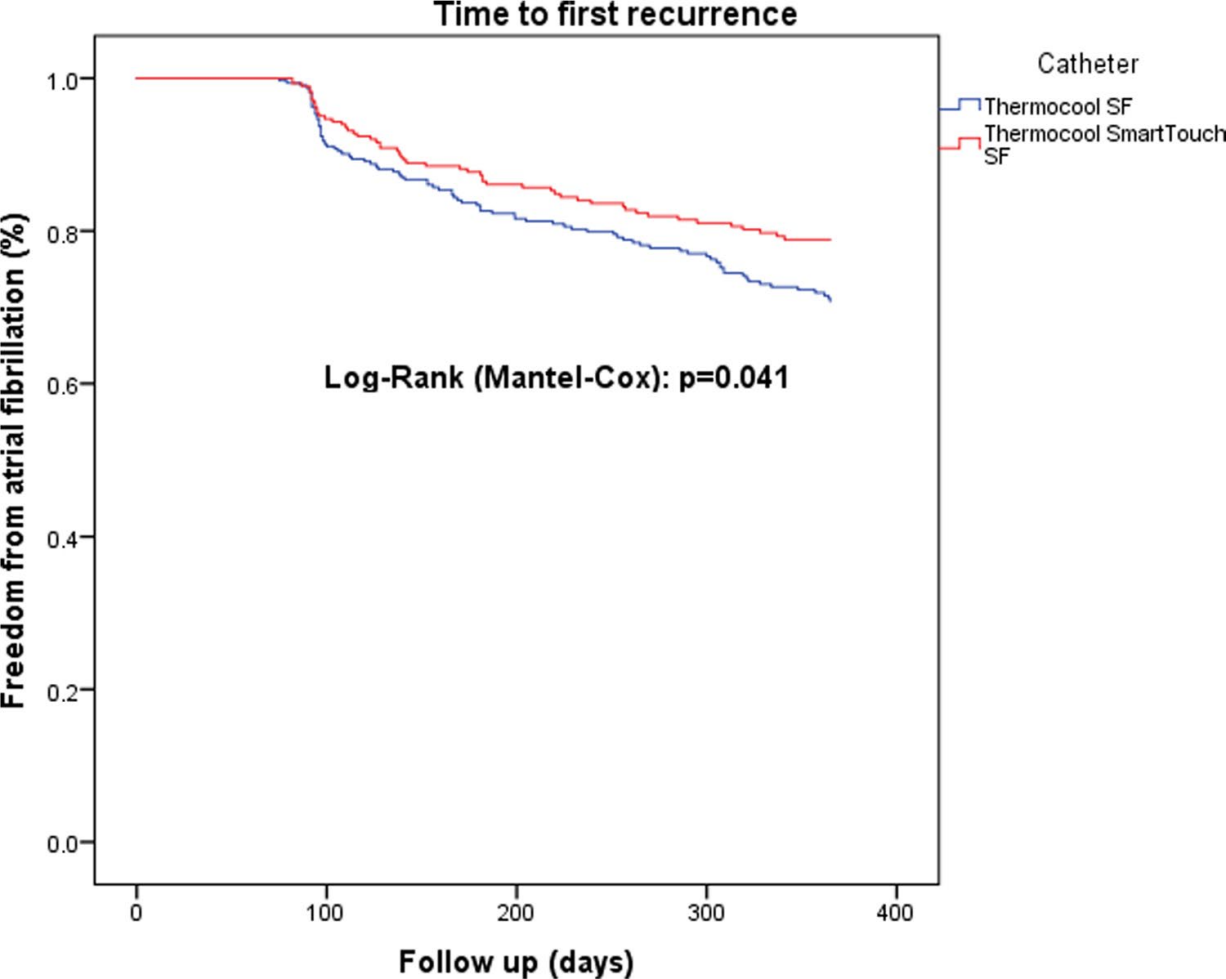
Time to first recurrence after last ablation

**Fig. 3:**
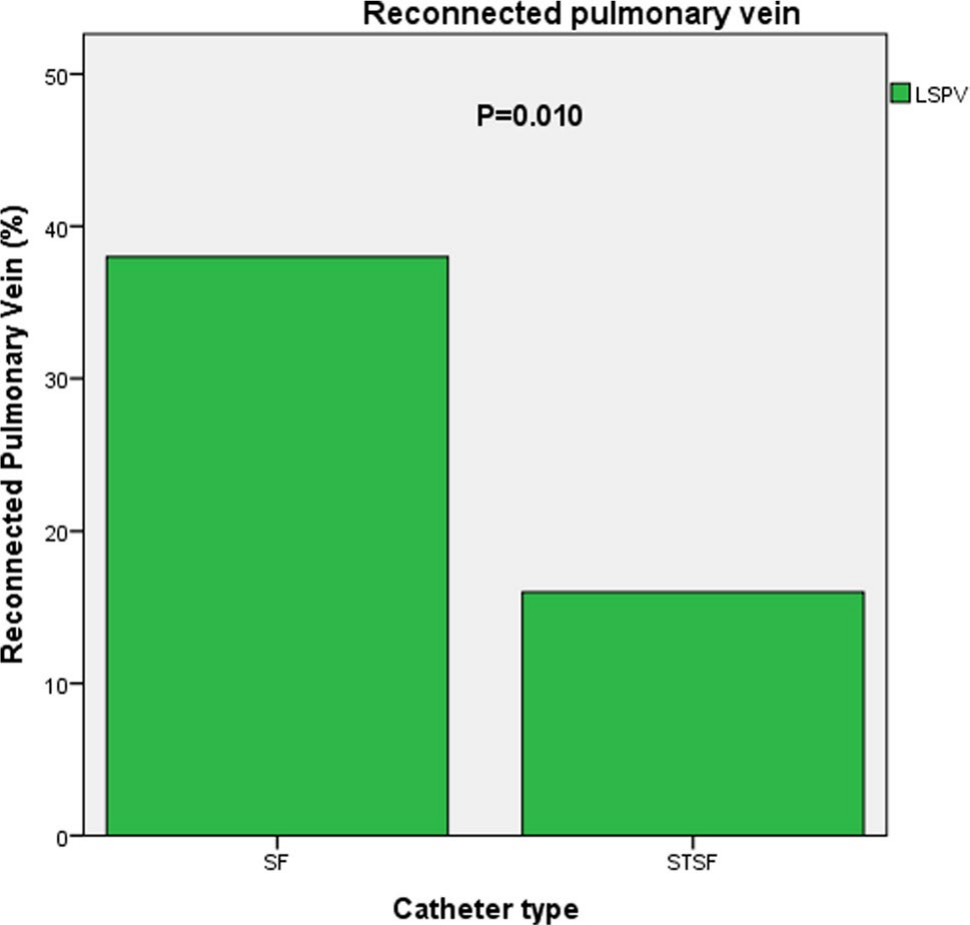
Percentage of reconnected left superior pulmonary vein

**Fig. 4:**
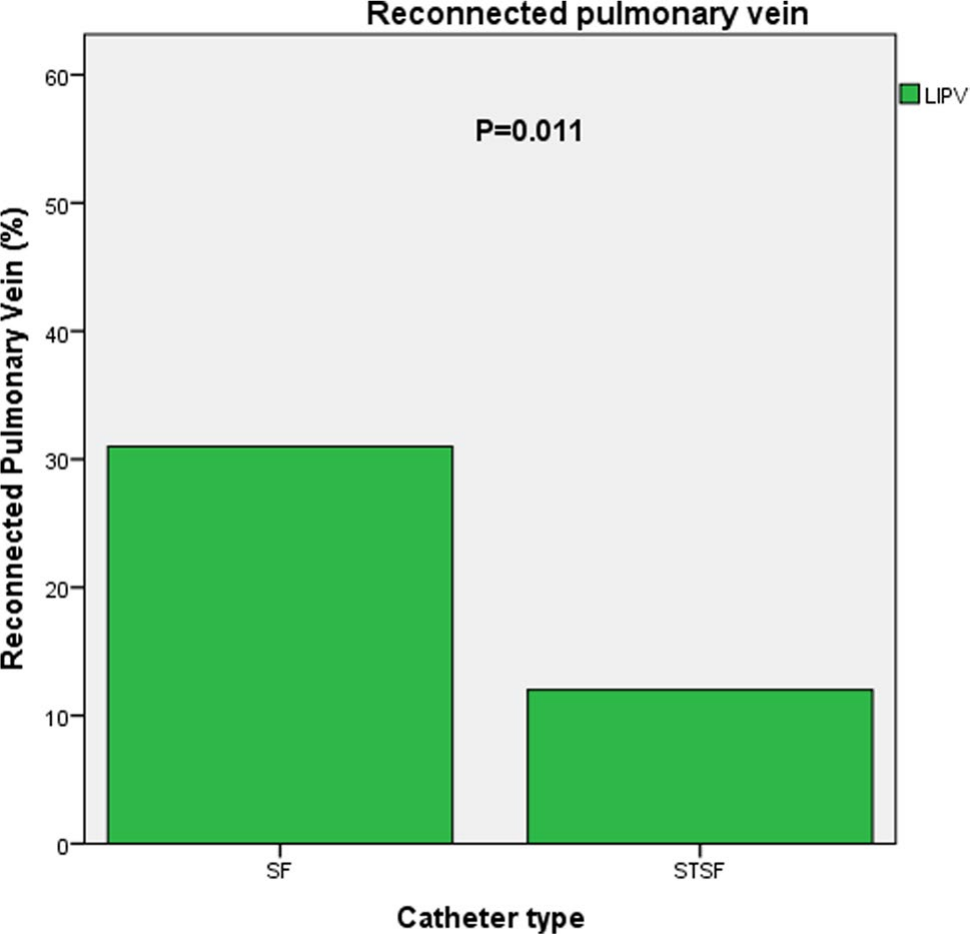
Percentage of reconnected left inferior pulmonary vein

Time to first recurrence after any ablation procedure differed not significantly between the CF group and non-CF group (*P* = 0.084, Fig. 1). However, after correction to the last available procedure, significantly more patients were in sinus rhythm after 1 year in the CF group (*P* = 0.041, Fig. 2). A multivariable Cox regression analysis was calculated to define predictors of AF recurrence after the procedure. Non-CF ablation, age, persistent AF were associated with a higher risk of recurrence of AF (Table 4). The analysis of repeat ablations showed a marginal non-significance between the mean number of reconnected veins (non-CF: 1.63 ± 1.3 vs. CF 1.25 ± 1.2; *P* = 0.067), however significantly higher rate of reconnections by the left superior and left inferior pulmonary veins after non-CF ablation (*P* < 0.001, Figs. 3, 4).

**Table 4:** Proportional hazard analysis for primary endpoint

Parameter	Univariate analysis			Multivariate analysis		
	HR	*P* value	95% CI	HR	*P* value	95% CI
Age	1.031	** < 0.001**	1.016–1.047	1.024	** < 0.001**	1.011–1.037
Persistent AF	1.712	**0.001**	1.248–2.349	1.484	**0.003**	1.142–1.931
Non-CF catheter	1.397	**0.025**	1.043–1.872	1.326	**0.020**	1.046–1.682
LA diameter	1.027	**0.012**	1.006–1,049	1.012	0.156	0.995–1.030
COPD	1.642	0.072	0.956–2.820			
Diabetes	1.439	0.080	0.958–2.161			
Female sex	1.288	0.095	0.957–1.732			
DCM	1.671	0.168	0.805–3.471			
Cardioversion	1.245	0.234	0.868–1.787			
BMI	0.986	0.359	0.958–1.016			
Hyperlipidemia	1.144	0.363	0.856–1.529			
CAD	1.101	0.583	0.782–1.550			
LVEF	0.995	0.735	0.970–1.022			
Smoking	1.054	0.778	0.731–1.521			
Sleep Apnea	1.050	0.861	0.609–1.810			
Hypertension	1.008	0.966	0.701–1.448			

*AF*, atrial fibrillation; *CF*, contact force; *LA*, left atrial; *COPD*, chronic obstructive pulmonary disease; *DCM,* dilatative cardiomyopathy; *BMI,* body mass index; *CAD*, coronary artery disease; *LVEF*, left ventricular ejection fraction

## Discussion

### Main finding

The main finding of this retrospective observational study is a significantly lower 1-year AF recurrence rate of CF-guided PVI after the last procedure compared to the non-CF-guided approach in a real-life cohort of AF patients. To the best of our knowledge, our study is the largest cohort of patients with the most ablation cases comparing the same irrigated tip ablation catheter with or without using contact force–sensing technology, showing a significant difference in 1-year success after adjusting for multiple confounders of PVI success rates. Moreover, our findings are also supported with the analysis of pulmonary vein reconnections by the redo ablations. Importantly, compared to previous studies which included a majority of patients with paroxysmal AF, most of our patients presented with persistent AF.

### Literature overview

Feasibility of CF-guided PVI in AF has been accompanied with continued debate in the light of previous conflicting RCTs and OSs [13]. Comparisons of CF-assisted PVI with non-CF PVI were often conducted in rather small patient groups with a majority of patients presenting with paroxysmal AF without multivariate statistical analysis [21–25]. Various studies compared different catheter technologies or older catheter types to CF-guided PVI [22, 25–29]. Recent meta-analyses included all available studies to date [11–13]. Most of these studies did not find a significant difference between CF PVI and non-CF PVI. Based on the available data, Virk et al. questioned if previous OSs would be conclusive in the light of RCTs on the effectiveness of CF-guided PVI [30]. Only one previous OS presented the same large number of cases with mainly persistent AF. Jarman et al. presented a retrospective case–control data showing CF catheter independently predicted clinical success in ablating paroxysmal AF, but not persistent AF in a multivariate analysis [31]. For this data series, 600 cases (200 CF, 400 non-CF) were followed for 11.4 ± 4.7 months in paroxysmal AF and 10.4 ± 4.5 in persistent AF. Although this study included a large case cohort, there were several significant differences at baseline, including a small difference in follow-up duration between the CF and non-CF groups. The only randomized study comparing CF versus non-CF by persistent atrial fibrillation was conducted by Conti et al. [20]. The TOUCH-AF randomized trial was conducted in a one-to-one manner, randomizing patients to a CF-guided versus CF-blinded strategy. It is important to mention that this ablation strategy included a wide antral pulmonary vein isolation plus a mandatory roof line. Patients were followed for 12 months. Single procedure success was 60% in the CF-guided arm and 63% in the CF-blinded arm off drugs. Lesions with gaps were associated with significantly less force.

### Efficacy endpoint

In the context of possible explanations for the conflicting previous data, several clinicals and a statistical factor should be considered. As a possible explanation, circumferential continuous ablation lines aim to electrically disconnect the PV antrum from the body of the LA. In cases, where these goals are not achieved, PVs are either partly or remain only temporarily isolated, leading to AF recurrence. The association between CF ablation and improved arrhythmia outcomes are consistent with the findings of a previous study in which the use of CF significantly reduced the incidence of acute PV reconnection following a 1-h waiting period [32]. Makimoto et al. found lower CF values during point-by-point left atrial mapping, the lowest CF obtained at left pulmonary veins [33]. This finding was in line with our analysis of pulmonary vein reconnection, showing significantly more reconnections at the left pulmonary veins. In our experience, the lowest CF values seemed to be present during the ablation of the ridge between the left pulmonary veins and the left atrial appendage. Therefore, this location might be predestined for reconnections. This finding is also supported by the literature [34].

In addition, avoiding ablation with suboptimal CF may reduce late development of gaps within linear lesions with resultant loss of clinical benefit and risk of proarrhythmia [34]. In persistent AF, while gaps in PV lesions are an important mechanism of recurrence, other mechanisms are also crucial, demonstrated most strikingly by the ability of AF to persist after PV ablation in some cases. The more advanced atrial substrate may often fibrillate in the presence of intact lesions. We could presume that non-CF ablation was lacking efficacy in creating continuous additional LA linear ablation lesions due to unknown force applied, making patients more prone to atrial fibrillation or even more to atrial tachycardia recurrence in persistent atrial fibrillation patients.

Previous OSs and their meta-analysis showed a significant efficacy benefit by paroxysmal AF; however, these findings did not translate to persistent AF, mainly because of lacking data in patients with persistent AF. A present meta-analysis of randomized controlled data is a critical addition to literature evaluating the safety and efficacy of CF-sensing AF ablation [13]. The lack of positive effect in RCTs, as compared to OS, have been attributed to low sample size and lack of statistical power [13]. Furthermore, most included RCTs used CF catheters in both arms, whereas OS compared CF catheters to already available catheters. It is possible that other properties of these newer catheters (e.g., stiffness) improved outcomes in the control group in RCTs, despite operators being blinded to CF data [13]. Analyzing our CF ablations, we did not find a significant difference between the first 193 and last 193 ablations regarding the 1-year procedural success (log-rank [Mantel-Cox] *P* = 0.833). To exclude any additional confounders, a Cox regression with backward elimination was performed. Experience with CF catheters did not influence the 1-year AF/AT-free survival (*p* = 0.868; HR = 0.968; 95% CI 0.661–1.419) in our collective.

Finally, it is important to mention that force time integral was proven to be less predictive of durable ablations lesions compared to the ablation index formula [35]. These findings are not incorporated in this study because patient recruitment was closed before ablation index was established in our center. Furthermore, ablation index is not the only factor in the creation of durable PVI lesions. The stability of the ablation catheter and interlesion distance index also plays a crucial role in effective PVI as it was proven in the CLOSE study [36]. Our study aimed at an interlesion distance of ≤ 6 mm; however, catheter stability as a controllable factor through Carto Visitag® Module was not yet available during our study.

### Procedural endpoint

In line with the aforementioned meta-analysis [13], the use of CF-sensing catheters in the present investigation was associated with considerably shorter fluoroscopy and total procedure times. However, in previous subgroup analysis of RCTs, neither procedure nor fluoroscopy duration were significantly reduced by CF guidance. According to Virk et al., this may be explained by operators receiving real-time CF feedback spending extra time calibrating CF to the target value prior to each point-by-point ablation, thereby counteracting potential time saved from the reduced need to address acute reconnections, or operator adaption with greater exposure to CF technologies and its associated learning curve [13]. After the analyses of the first 193 CF ablations and comparing them to the last 193 ablations, however we did not find significant difference between the total procedural time (110 ± 30 vs. 114 ± 32 min; *P* = 0.185).

### Safety endpoint

The complication rates in our study data are comparable to those in worldwide surveys and previous meta-analyses [12, 13, 37]. Theoretically, procedural safety should be an undisputed advantage of CF ablation: it is intuitively expected that the use of real-time CF feedback during ablation may protect against steam pops and perforations occurring due to excessive CF [5]. In our patient cohort, the difference in the major complication rate failed to reach statistical significance between groups. The use of CF guidance did not reduce the number of pericardial effusions, as it was also seen in pooled previous data [13]. However, it is interesting to note that the incidence of phrenic nerve palsy was higher for CF-guided ablations (2 cases) compared to non-CF ablation (0 case). We hypothesize that the use of CF led to a continuous higher applied force in the anterior area of the right superior and right inferior pulmonary vein leading to increased incidence of this complication.

### Limitations

This was a non-randomized single-center observational study. AF recurrence rates were to some extent dependent on the patient’s and general practitioner’s awareness and responsiveness. Thus, asymptomatic episodes of AF may have been missed. The same experienced operators performed all of our procedures. However, as procedural experience increases, operators develop more skill and experience which may lead to improved outcomes. We cannot fully exclude that this aspect plays also a role. Lastly, our study did not incorporate ablation index or Carto Visitag® module with stability settings as these technical improvements were not readily available in our center at the time of this study.

## Conclusions

In this large observational cohort with a 12-months follow-up, pulmonary vein ablation using a contact force–guided catheter results in a lower AF recurrence rates and improved procedural time and total fluoroscopy time compared to non-contact force–guided catheter ablation. Complication rates did not significantly differ between non-contact force–guided ablation compared to contact force–guided ablation.

## Data Availability

Data available on request due to privacy/ethical restrictions.
